# Long-Term Chili Monoculture Alters Environmental Variables Affecting the Dominant Microbial Community in Rhizosphere Soil

**DOI:** 10.3389/fmicb.2021.681953

**Published:** 2021-07-01

**Authors:** Wenjing Chen, Xiaodong Guo, Quanen Guo, Xuelian Tan, Zhigang Wang

**Affiliations:** ^1^Institute of Soil, Fertilizer and Water-Saving Agriculture, Gansu Academy of Agricultural Sciences, Lanzhou, China; ^2^College of Life Sciences, Agriculture and Forestry, Qiqihar University, Qiqihar, China; ^3^Heilongjiang Provincial Technology Innovation Center of Agromicrobial Preparation Industrialization, Qiqihar, China; ^4^Key Laboratory of Efficient Utilization of Water in Dry Farming, Lanzhou, China

**Keywords:** chili, continuous cropping, rhizosphere microorganisms, dominant microbial community, environmental filtering

## Abstract

Continuous cropping negatively affects soil fertility, physicochemical properties and the microbial community structure. However, the effects of long-term chili monoculture on the dominant microbial community assembly are not known. In this study, the impact of long-term chili monoculture on the correlation between the dominant microbial community and soil environmental variables was assessed. The results indicated that increasing duration of chili monoculture generated significant changes in soil nutrients, soil aggregates and soil enzymes: nutrient contents increased overall, mechanically stable macroaggregates increased and microaggregates decreased, water-stable macroaggregates and microaggregates decreased, β-glucosidase decreased nonlinearly, and nitrate reductase and alkaline phosphatase activities showed a nonlinear increase. Moreover, an increasing number of years of chili monoculture also affected the structure of the dominant microbiota, with substantial changes in the relative abundances of 11 bacterial and fungal genera. The drivers of the dominant microbial community assembly in rhizosphere soil were soil moisture, abiotic nitrogen, pH and salt.

## Graphical Abstract

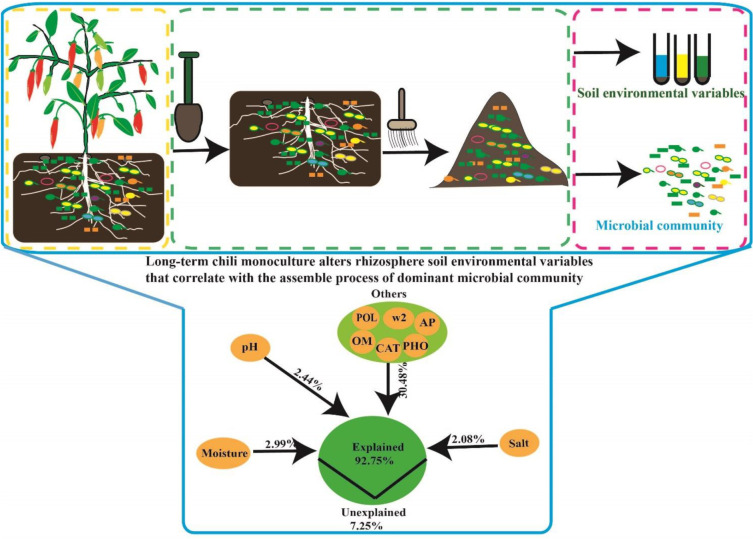

## Introduction

The rhizosphere, the narrow zone of soil that surrounds and is influenced by plant roots, is home to a vast number of microorganisms (Philippot et al., 2013). Rhizosphere microorganisms have a significant effect on root biology and plant growth, nutrition, and development (Li et al., 2017). The majority of microbial communities are composed of a few abundant taxa and a large number of rare species, and abundant microbes account for the majority of biomass and carbon cycling in ecosystems (Wu et al., 2017; Jiao and Lu, 2020). Thus, revealing this community component is crucial to understanding ecosystem function.

Chili (*Capsicum annuum* L.), a vital horticultural crop, is commonly used as a condiment to flavor cooked food and has been used by cultures around the world since 7000 BC (Liu et al., 2012). More than 200 constituents have been identified in chili, and some of its active constituents play numerous beneficial roles in human health (Varghese et al., 2017), while chili monoculture also fosters insect and pathogenic microbes (Eric, 2010). Therefore, revealing the effect of long-term chili monoculture is critical to determining the links between community stability and ecosystem function.

The effects of continuous cropping of different plants have been reported. Continuous potato cropping caused a decline in soil fertility and an increase in pathogenic microbes and aggravated the toxic effects of root secretions (Qin et al., 2017). Additionally, long-term continuous monocultures of tomato and tea bush decreased soil enzymatic activities, microbial metabolic activity, and microbial biomass and caused a shift in the microbial community composition and structure (Fu et al., 2017; Li et al., 2017). Furthermore, continuous cropping of strawberries disturbed the soil food web, and the general health of the soil deteriorated, with especially negative effects on soil fertility and physicochemical properties, leading to a decline in crop productivity (Li et al., 2016). Most plants require microbial symbiosis for a more efficient uptake of nutrients from the soil (Selvakumar et al., 2018). Therefore, understanding the effects of long-term chili monoculture on the soil are critical to discovering obstacles to continuous cropping.

Environmental filtering is a key determinant of species distribution and abundance (Jiao and Lu, 2020). For example, the dominant drivers of the soil diazotrophic community structure are soil pH, soil organic matter, soil moisture, and the soil carbon:nitrogen ratio (Fan et al., 2018). Additionally, pH has a critical role in the biogeographic distribution of terrestrial bacteria (Bahram et al., 2018; Tripathi et al., 2018). Moreover, the variation in soil organic matter could alter the relative influences of different assembly processes in shaping soil bacterial communities (Feng et al., 2018). Soil microbial communities strongly influence ecosystem function such that predicting function may rely on understanding ecological processes that assemble communities (Feng et al., 2018). Therefore, we hypothesized that soil environmental factors were changed by long-term chili monoculture and regulated the community assembly of abundant microbes in rhizosphere soil. Moreover, chili disease was observed in the field but was relieved after several years of continuous planting, thus we expected that long-term chili cultivation would lead to accumulation of beneficial microorganisms or pathogenic microorganisms, and focused on the changes of the soil dominant microbial community in the field of chili growth promotion and disease resistance were important for chili production.

Belowground microbial communities strongly influence ecosystem function such that predicting function may rely on understanding ecological processes that assemble communities. Uncertainty remains, however, in what governs the relative contributions of different ecological processes. To help fill this knowledge gap, we test the general hypothesis that both initial state and degree of change in environmental conditions govern the relative contributions of different ecological assembly processes.

In this study, the rhizosphere soil of chili under various years of continuous cropping was used as a research material to clarify the dominant microbial community assembly and determinants of the dominant microbial community dynamics by studying soil physical and chemical properties, soil enzyme activities and high-throughput sequencing, the results of which provide novel insights for the targeted research of microbial agents and regulation the properties of soil that could improve soil continuous cropping barrier and enhance the function of soil biological systems.

## Materials and Methods

### Study Site and Sample Collection

The solar greenhouse was established in Gansu Province, China (37°53′15″N, 102°40′50″E), in a cropping system of continuous chili. The area has a typical continental climate, with an annual average temperature of 7.6°C and precipitation of 170 mm. The soil was desert soil and comprised 25% sand, 14% silt, and 11% clay. The experimental soil samples were collected from four solar greenhouses containing the same chili cultivar that were continuously for 1, 5, 10, and 20 years. The 4 solar greenhouses about 500 m^2^ in size. The agronomic management and fertilization regime were similar in the four solar greenhouses, and annual NPK fertilization comprised 120 kg/ha as urea, 75 kg/ha as calcium magnesium phosphate, and 90 kg/ha as potassium chloride. For each solar greenhouse, 10 chili plants were randomly selected, and the whole plant was dug with a shovel at a depth of 20 cm and a width of 30 cm, respectively. The plants were shaken vigorously to remove soil in the root zone. Then collected the soil within 1–2 mm of the root with the brush and combined to form one composite soil sample per plot. Soil samples were put into sterile plastic bags, and then divided into two subsamples and stored at either 4°C for soil geochemical variable measurements or at –80°C for microbial community analysis.

### Analysis of Soil Chemical Properties

Soil moisture was measured by the oven-drying method (Fan et al., 2018), and soil pH was determined by a pH meter (Sartorius Basic pH meter PB-10, Goettingen, Germany) with a soil/water ratio of 1:2.5 (Liu et al., 2014b; Jiao et al., 2019). The soil salt content was determined by the sum of cations and anions with a soil/water ratio of 1:5 (Li et al., 2008). The concentrations of organic matter, hydrolyzable nitrogen, and soil available phosphorus were measured using potassium dichromate oxidation, alkaline digestion-diffusion and sodium hydrogen carbonate solution-Mo–Sb anti spectrophotometry methods, respectively, according to the Agricultural Industry Standards of the People’s Republic of China (NY/T1121.6-2006, LY/T1229-1999, and HJ704-2014). The concentration of soil available potassium was determined by a flame spectrophotometer (AA320N, Shanghai Precision Instrument Co., Ltd., Shanghai, China), and soil ammonium nitrogen and soil nitrate nitrogen were determined on a continuous-flow auto analyzer (PROXI-MA, Alliance Instruments, Paris, France). Each sample performed in triplicate for soil chemical properties.

### Analysis of Soil Aggregates and Enzyme Activities

The distributions of water-stable aggregates and mechanically stable aggregates were obtained from a 50 g soil sample using the wet sieving and dry sieving, respectively (Garey, 1954; Puget et al., 2000; Liu et al., 2014a; Lin et al., 2019). Four aggregate classes (>2, 1–2, 0.25–1, and <0.25 mm) were obtained with sieve set of 2, 1, and 0.25 mm. Briefly, soil sample was put on the first sieve of the set in a water bucket and was gently moistened for 10 min. The aggregates were separated by moving the sieve vertical with a speed of 30 rpm for 5 min after pre-wetting. All aggregate-size fractions remaining on each sieve were collected, dried and weighed; for mechanically stable aggregates, 50 g soil sample was put on the first sieve of the set, the aggregates were separated by moving the sieve vertical with a speed of 30 rpm for 5 min. Each sample performed in triplicate for aggregate.

The activities of catalase, β-glucosidase, nitrate reductase, polyphenol oxidase, urease and alkaline phosphatase were determined using a soil catalase assay kit (Product No. QS2937, Shanghai Cablebridge Biotechnology Co., Ltd., Shanghai, China), a soil β-glucosidase assay kit (Product No. BC0160, Beijing Solarbio Biotechnology Co., Ltd., Beijing, China), a soil nitrate reductase assay kit (Product No. QS2938, Shanghai Cablebridge Biotechnology Co., Ltd., Shanghai, China), a soil polyphenol oxidase assay kit (Product No. QS2934, Shanghai Cablebridge Biotechnology Co., Ltd., Shanghai, China), a soil urease assay kit (Product No. QS2933, Shanghai Cablebridge Biotechnology Co., Ltd., Shanghai, China), and a soil alkaline phosphatase assay kit (Product No. BC0280, Beijing Solarbio Biotechnology Co., Ltd., Beijing, China), respectively. Each sample performed in triplicate for enzyme activity.

In brief, 0.2 g of soil was treated with 5 mL of extraction solution for 20 min, and the reaction mixtures were used to determine the activities of the six enzymes using the appropriate assay kits. For catalase activity, the mixture was centrifuged at 10,000 rpm for 10 min at 4°C, and the supernatants were used to determine the activities of catalase activity by measuring the absorbance at 240 nm using a UV 1100 spectrophotometer (Mei puda, Beijing, China) as an indicator of the decrease of H_2_O_2_ (Li and Schellhorn, 2007); for β-glucosidase activity, the reaction mixtures were heated in a boiling water bath for 5 min after incubated for 1 h in a water bath at 37°C. The mixture was centrifuged for 10 min at 10,000 rpm and the absorbance measured at 410 nm (Turner et al., 2002); for nitrate reductase, the reaction mixtures were incubated for 24 h in a water bath at 37°C. The mixture was centrifuged for 5 min at 8,000 rpm and the absorbance measured at 520 nm (Singh and Kumar, 2008); for polyphenol oxidase, the reaction mixtures were incubated for 1 h at 37°C. The mixture was centrifuged for 10 min at 10,000 rpm and the absorbance measured at 410 nm (Adamczyk et al., 2009); for urease, the mixture was centrifuged at 10,000 rpm for 10 min, and the supernatants were used to determine the activities of catalase activity by measuring the absorbance at 690 nm (Singh and Kumar, 2008); for alkaline phosphatase, the reaction mixtures were incubated for 24 h at 37°C. The mixture was centrifuged for 10 min at 10,000 rpm and the absorbance measured at 398 nm (Sardans et al., 2008). Enzyme activity was calculated by using a standard curve.

According to the operating instructions of the assay kits (Shanghai Cablebridge Biotechnology Co., Ltd., Shanghai, China), the activities of catalase (Eq. 1), β-glucosidase (Eq. 2), nitrate reductase (Eq. 2), polyphenol oxidase (Eq. 2), urease (Eq. 2) and alkaline phosphatase (Eq. 3) were calculated based on Eqs 1–3 below:

(1) $$\begin{array}{c} E\left(\text{U}/\text{g}\right)=\frac{\left(An-Am+As\right)\times V}{\varepsilon \times m\times T} \end{array}$$

(2) $$\begin{array}{c} E\left(\text{U}/\text{g}\right)=\frac{C\times V}{m\times T} \end{array}$$

(3) $$\begin{array}{c} E\left(\text{U}/\text{g}\right)=\frac{\left[Ct\times \left(Am-Ab\right)÷\left(At-Ab\right)\times V\right]}{m\times T} \end{array}$$

Where *E* is enzyme activity (U/g), U is the enzyme activity unit, *An* is the absorbance of soilless sample, *Am* is the absorbance of sample, *As* is the absorbance of no substrate sample, *Ab* is the absorbance of blank tube, *At* is the absorbance of tube standard, *V* is the volume of the reaction system, ε is the molar extinction coefficient of H_2_O_2_ (43.6 L/mol/cm), *m* is the quality of the soil sample, *C* is the concentration of the sample, *Ct* is the concentration of standard liquid, and *T* is the reaction time.

### High-Throughput Sequencing and Bioinformatics Analysis

Three replicates of each sample were used to extract soil DNA from a 0.5 g soil sample using the Powersoil^®^ DNA Isolation Kit (MoBio Laboratories, Carlsbad, CA, United States), purified using a DNA purification kit (DP209, Tiangen Biotechnology Co., Ltd, Beijing, China) according to the manufacturer’s instructions, and analyzed for concentration and quality using a Nanodrop ND-2000 spectrophotometer (Nano Drop Technologies, Wilmington, DE, United States). Then, the integrity of the extracted DNA was determined with 0.8% agarose gel electrophoresis (Li et al., 2020).

To amplify the V3–V4 region and ITS region of the bacterial and fungal genes, the universal primers 338F (5′-ACTCCTACGGGAGGCAGCA-3′) and 806R (5′-GGACTACH VGGGTWTCTAAT-3′) as well as ITS1F (5′-CTTGGTCA TTTAGAGGAAGTAA-3′) and ITS1R (5′-GCTGCGTTCT TCATCGATGC-3′) were used in PCR, and the detailed PCR amplification procedure was reported previously (Wagg et al., 2019). Then, the integrity of the PCR products was determined with 1.8% agarose gel electrophoresis. Finally, DNA sequencing results were obtained using the Illumina MiSeq platform (San Diego, CA, United States).

The original DNA sequences were generated using Quantitative Insights into Microbial Ecology (QIIME, version 1.17) software to generate valid sequences, and then the high-quality sequences were clustered into operational taxonomic units (OTUs) based on a 97% similarity level using the UPARSE algorithm (version 7.1^1^). Moreover, the chimeric sequences were identified and removed using UCHIME. Finally, 16S and ITS OTU sequences were taxonomically assigned using the Ribosomal Database Project (RDP^2^) Bayesian classifier at a 70% threshold against the SILVA and UNITE databases and then identified to the phylum, class, order, family and genus levels (Wagg et al., 2019).

### Statistical Analyses

The effects of continuous cropping on the soil chemical and biochemical properties and enzyme activities as well as on the soil microbial community structure were assessed using linear regression analysis with the Pearson method. Most of the indicators were non-linearly correlated with continuous cropping years in this study, it’s not precise in described the data structure with linear regression analysis, so the nonlinear response to continuous cropping was further analyzed using locally estimated scatterplot smoothing (LOESS) regression (Goh et al., 2017; Zhang et al., 2020). LOESS is a nonparametric regression method that uses weights to adjust the fit in a localized fashion along points in a scatterplot, and reduces the effects of outliers while retaining general trends, thus it more flexible to fitting a line to data. Additionally, the objective of LOESS is to confirm the best fits for segments of the data, which generally utilizes a parabola or higher-order polynomial instead of a straight line to fit data, thus LOESS is the best way to described the data structure (Wegmueller et al., 2021). Then, the relative influence of the environmental variables on the dominant microbial community was determined using the aggregated boosted tree (ABT) method (Death, 2007). Moreover, variance partitioning analysis (VPA) was used to determine the contribution of the environmental variables to the dominant microbial community (Ren et al., 2019). Additionally, the correlation between soil physical and chemical properties and dominant strains was assessed with the Mantel test (Li et al., 2021). Finally, one-way ANOVA followed by Tukey’s HSD test was performed to compare the response of the univariate abiotic and biotic parameters among the different continuous cropping levels, with *p* < 0.05 denoting significance. All analyses were performed in R software.

## Results

### Chemical Characterization of the Soil

**FIGURE 1:**
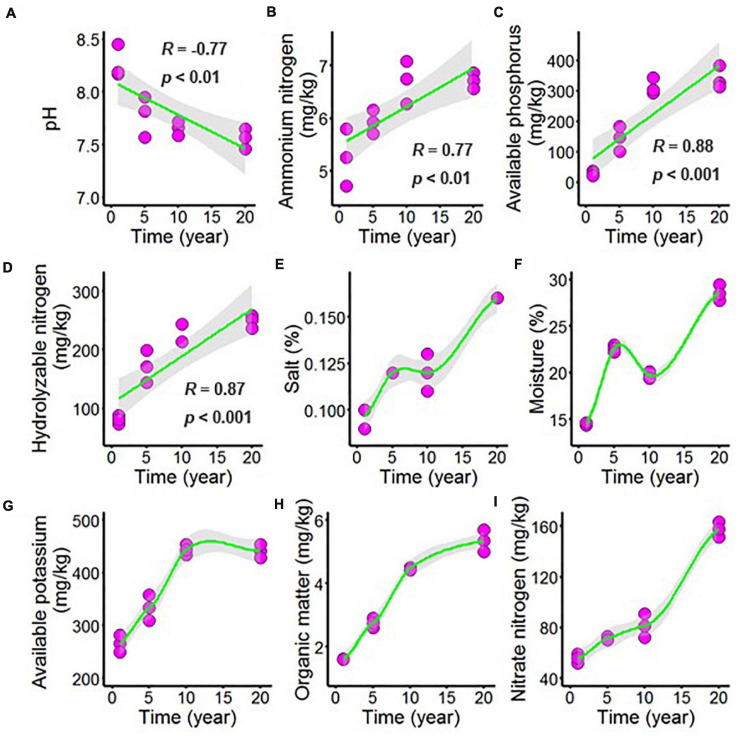
Effect of long-term chili monoculture on the chemical properties of rhizosphere soil. Long-term chili monoculture altered the pH **(A)**, ammonium nitrogen **(B)**, available phosphorus **(C)**, hydrolyzable nitrogen **(D)**, salt **(E)**, moisture **(F)**, available potassium **(G)**, organic matter **(H)**, and nitrate nitrogen **(I)**. Soil chemical properties show different patterns of response to continuous cropping duration (1, 5, 10, and 20 years), including linear and nonlinear relationships. Solid lines represent linear or locally estimated scatterplot smoothing (LOESS) fits, and gray areas denote the 95% confidence intervals.

Long-term chili monoculture altered the chemical properties of the rhizosphere soil, including changing the concentrations of organic matter (OM), available phosphorus (AP), available potassium (AK), hydrolyzable nitrogen (HN), ammonium nitrogen (AN), and nitrate nitrogen (NN), as well as the pH, salt and moisture. In Figure 1 and Supplementary Table 1, increasing continuous cropping duration was negatively correlated with pH (Figure 1A, *R* = –0.77, *p* < 0.01) and positively correlated with AN (Figure 1B, *R* = 0.77, *p* < 0.01), AP (Figure 1C, *R* = 0.88, *p* < 0.001) and HN (Figure 1D, *R* = 0.87, *p* < 0.001). However, the minority of the chemical parameters demonstrated nonlinear responses to continuous cropping duration, including the content of salt in chili rhizosphere soil (Figure 1E), which increased as continuous cropping levels reached 5 years and then remained stable but increased again after 10 years; the moisture of the chili rhizosphere soil samples (Figure 1F) continuously increased with continuous cropping for 5 years, then decreased at 10 years, and then increased again. The AK content (Figure 1G) in the chili rhizosphere soil increased with fluctuations as continuous cropping reached 10 years, becoming stable, and the OM content (Figure 1H) increased with fluctuations. The NN content (Figure 1I) steadily increased as the continuous cropping duration reached 5 years and then remained stable, but after 10 years, NN in the chili rhizosphere soil samples increased sharply.

### Soil Aggregates

**FIGURE 2:**
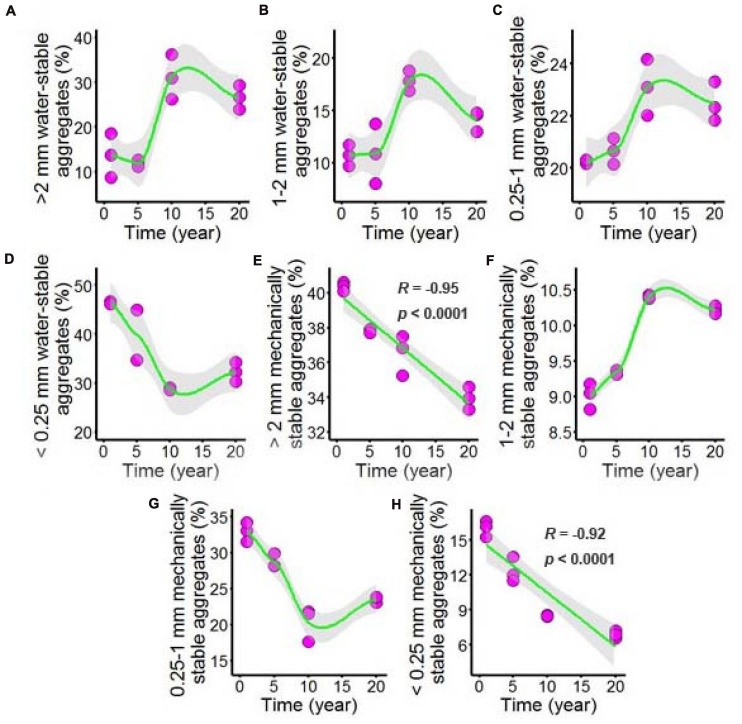
Effect of long-term chili monoculture on the aggregates of rhizosphere soil. Long-term chili monoculture altered >2 mm water-stable aggregates **(A)**, 1–2 mm water-stable aggregates **(B)**, 0.25–1 mm water-stable aggregates **(C)**, <0.25 mm water-stable aggregates **(D)**, >2 mm mechanically stable aggregates **(E)**, 1–2 mm mechanically stable aggregates **(F)**, 0.25–1 mm mechanically stable aggregates **(G)** and <0.25 mm mechanically stable aggregates **(H)**. Soil aggregates show different patterns of response to continuous cropping duration (1, 5, 10, and 20 years), including linear and nonlinear relationships. Solid lines represent linear or LOESS fits, and gray areas denote the 95% confidence intervals.

Both water-stable aggregates and mechanically stable aggregates of the chili rhizosphere soil samples were tested after long-term chili monoculture, and the results are shown in Figure 2 and Supplementary Table 2. The majority of the aggregates showed nonlinear responses to increasing duration of continuous cropping, including >2 mm water-stable aggregates (Figure 2A), 1–2 mm water-stable aggregates (Figure 2B) and 0.25–1 mm water-stable aggregates (Figure 2C), which remained stable as the continuous cropping duration reached 5 years, after which they sharply increased and then remained stable in the chili rhizosphere soil samples. The <0.25 mm water-stable aggregates (Figure 2D) had a totally different trend; the 1–2 mm mechanically stable aggregates (Figure 2F) increased as the continuous cropping duration reached 10 years, after which they remained stable in the chili rhizosphere soil samples. In contrast, the 0.25–1 mm mechanically stable aggregates (Figure 2G) decreased as continuous cropping reached 10 years, after which they remained stable in the chili rhizosphere soil samples. Increasing continuous cropping duration had a negative linear correlation with the >2 mm mechanically stable aggregates (Figure 2E, *R* = –0.95, *p* < 0.0001) and <0.25 mm mechanically stable aggregates (Figure 2H, *R* = –0.92, *p* < 0.0001).

### Soil Enzyme Activities

**FIGURE 3:**
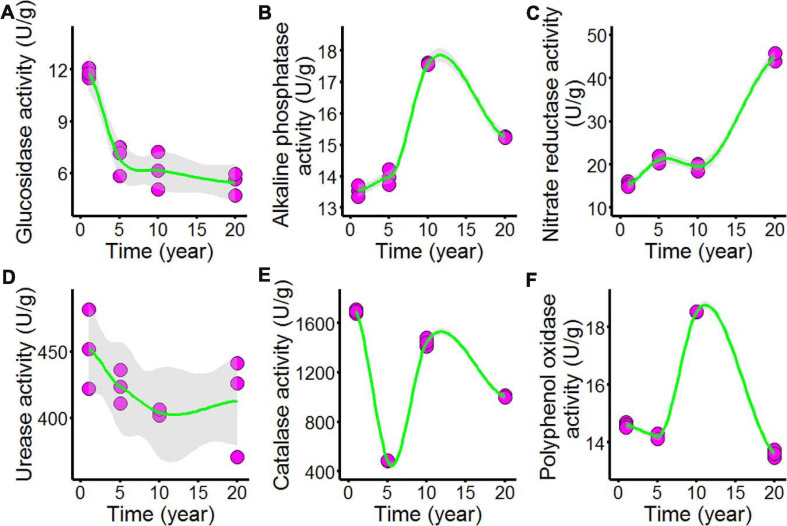
Effect of long-term chili monoculture on the enzyme activities of rhizosphere soil. Long-term chili monoculture altered the activities of μ-glucosidase **(A)**, alkaline phosphatase **(B)**, nitrate reductase **(C)**, urease **(D)**, catalase **(E)**, and polyphenol oxidase **(F)**. Soil enzymatic levels show different patterns of response to continuous cropping duration (1, 5, 10, and 20 years). Solid lines represent LOESS fits, and gray areas denote the 95% confidence intervals.

As shown in Figure 3 and Supplementary Table 3, we tested the enzyme activities of the chili rhizosphere soil samples, including the activities of β-glucosidase, nitrate reductase, urease, alkaline phosphatase, catalase, and polyphenol oxidase. All of the soil enzyme activities showed nonlinear responses to increasing continuous cropping duration: β-glucosidase (Figure 3A) declined as continuous cropping reached 5 years, after which it remained stable in the chili rhizosphere soil samples; alkaline phosphatase (Figure 3B) increased with fluctuations with continuous cropping for 5 years and then sharply increased at 10 years; after that, it decreased in the chili rhizosphere soil samples. Nitrate reductase (Figure 3C) increased as the continuous cropping duration reached 5 years and then remained stable; after 10 years, it sharply increased in the chili rhizosphere soil samples. The activity of urease (Figure 3D) was not significantly changed. The activities of catalase (Figure 3E) and polyphenol oxidase (Figure 3F) showed fluctuating changes.

### Soil Dominant Microbial Community Structure

**FIGURE 4:**
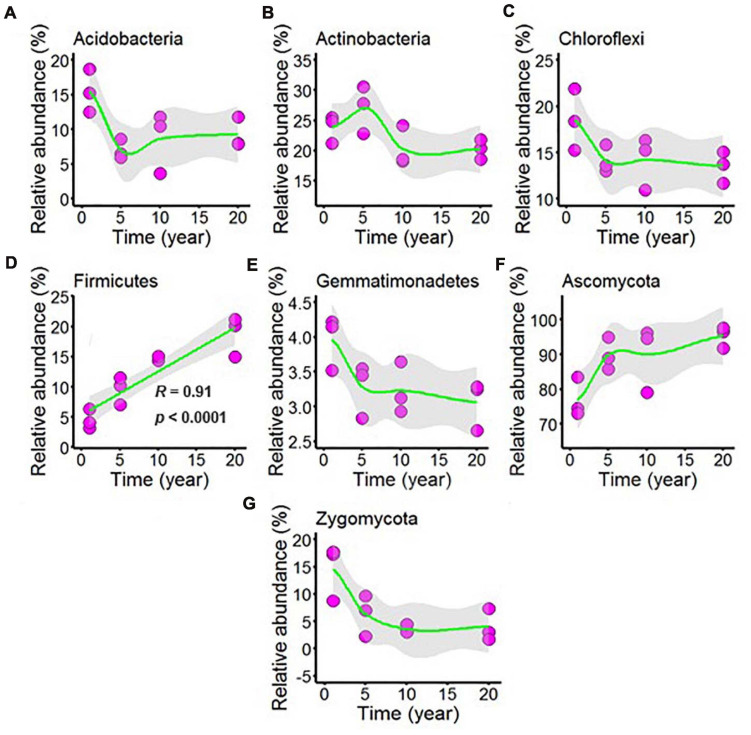
Effect of long-term chili monoculture on the dominant microbes at the phylum level in rhizosphere soil. Long-term chili monoculture altered the relative abundance of Acidobacteria **(A)**, Actinobacteria **(B)**, Chloroflexi **(C)**, Firmicutes **(D)**, Gemmatimonadetes **(E)**, Ascomycota **(F)**, and Zygomycota **(G)**.

Sequencing of the chili rhizosphere soil microbial communities generated a total of 577,777 bacterial sequences and 570,551 fungal sequences, and these sequences were clustered into 2,732 and 890 distinct OTUs for bacteria and fungi, respectively. The relative abundance exceeded 1% was considered to be the dominant microbes in all chili rhizosphere soil samples. The dominant bacteria was classified into phyla Acidobacteria, Actinobacteria, Chloroflexi, Firmicutes, and Gemmatimonadetes, and the dominant fungi phyla was Ascomycota and Zygomycota, and the dominant microbes was shown in Figure 4 and Supplementary Table 4. As shown in Supplementary Figure 4A, the relative abundance of Acidobacteria decreased with continuous cropping for 5 years and then remained stable; Increasing continuous cropping duration was positively correlated with Firmicutes (Figure 4D, *R* = 0.91, *p* < 0.0001); the relative abundance of Actinobacteria (Figure 4B), Chloroflexi (Figure 4C), Gemmatimonadetes (Figure 4E), Ascomycota (Figure 4F), and Zygomycota (Figure 4G) did not change at the levels examined.

**FIGURE 5:**
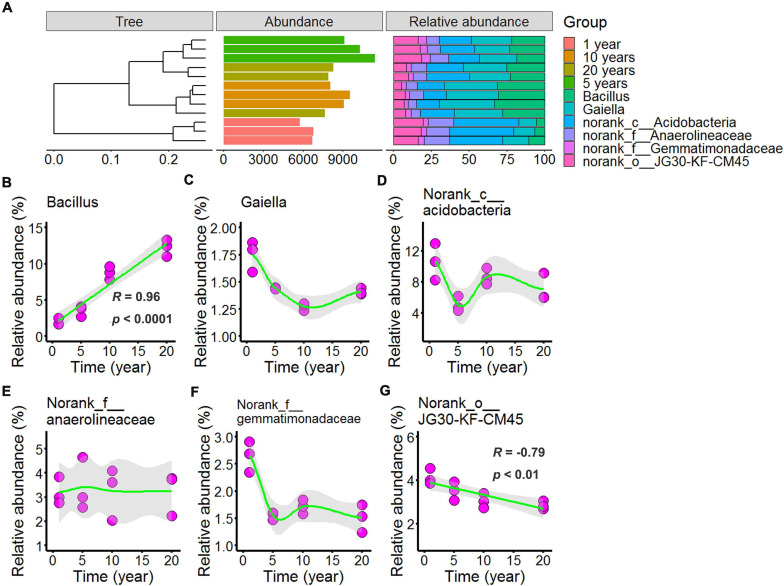
Effect of long-term chili monoculture on the dominant bacteria in rhizosphere soil. The “Abundance” in panel **(A)** was the total abundance of operational taxonomic units (OTUs). Long-term chili monoculture altered the relative abundance of Bacillus **(B)**, Gaiella **(C)**, norank_c__Acidobacteria **(D)**, norank_f__Anaerolineaceae **(E)**, norank_f__Gemmatimonadaceae **(F)**, and norank_○__JG30-KF-CM45 **(G)**. The relative abundances of dominant soil bacteria at the genus level of the taxonomic classification showed a nonlinear pattern in response to continuous cropping duration (1, 5, 10, and 20 years). Solid lines represent linear or LOESS fits, and gray areas denote the 95% confidence intervals.

**FIGURE 6:**
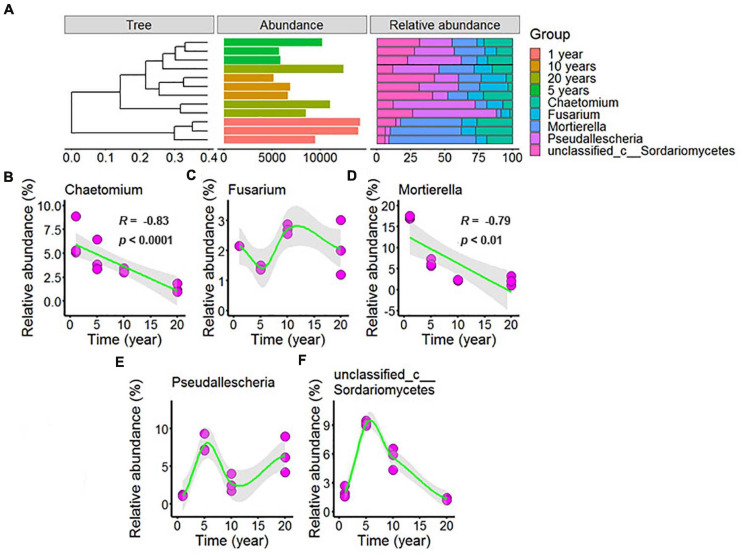
Effect of long-term chili monoculture on the dominant fungi in rhizosphere soil. The “Abundance” in panel **(A)** was the total abundance of OTUs. Long-term chili monoculture altered the relative abundance of Chaetomium **(B)**, Fusarium **(C)**, Mortierella **(D)**, Pseudallescheria **(E)**, and unclassified_c__Sordariomycetes **(F)**. The relative abundances of dominant soil fungi at the genus level of thetaxonomic classification showed different patterns of response to continuous cropping duration (1, 5, 10, and 20 years), including linear and nonlinear relationships. Solid lines represent linear or LOESS fits, and gray areas denote the 95% confidence intervals.

In Figures 5, 6 and Supplementary Tables 5, 6 showed the differences in the microbial community structure across long-term chili monocultures were investigated at the genus level. *Bacillus*, *Gaiella*, norank_c__Acidobacteria, norank_f__Anaerolineaceae, norank_f__Gemmatimonadaceae, and norank_o__JG30-KF-CM45 were the dominant bacteria (Figure 5A), of which the relative abundance exceeded 1% in all chili rhizosphere soil samples. As shown in Figure 5C, the relative abundance of *Gaiella* decreased with continuous cropping for 5 years and then remained stable; the relative abundance of norank_c__Acidobacteria (Figure 5D) decreased with continuous cropping for 5 years, then increased, and then showed no further changes in the chili rhizosphere soil samples; norank_f__Anaerolineaceae relative abundance (Figure 5E) did not change at the levels examined; norank_f__Gemmatimonadaceae (Figure 5F) decreased as the continuous cropping duration reached 5 years and then remained stable in the chili rhizosphere soil samples. Increasing continuous cropping duration was positively correlated with *Bacillus* (Figure 5B, *R* = 0.96, *p* < 0.0001) and negatively correlated with norank_o__JG30-KF-CM45 (Figure 5G, R = –0.79, *p* < 0.01).

Figure 6A showed that the dominant fungi were *Chaetomium, Fusarium, Mortierella, Pseudallescheria*, and unclassified_c__Sordariomycetes, and increasing continuous cropping levels correlated in a negative linear way with *Chaetomium* (Figure 6B, *R* = –0.83, *p* < 0.0001) and *Mortierella* (Figure 6D, *R* = –0.79, *p* < 0.01); the relative abundance of *Fusarium* (Figure 6C) decreased with continuous cropping for 5 years, then increased, and 10 years and 20 years showed no significant change with 1 year in the chili rhizosphere soil samples; the relative abundance of *Pseudallescheria* (Figure 6E) showed fluctuating changes, and 20 years showed no significant change with 5 years in the chili rhizosphere soil samples; the relative abundance of unclassified_c__Sordariomycetes (Figure 6F) increased with continuous cropping for 5 years, then decreased, and 20 years showed no significant change with 1 year in the chili rhizosphere soil samples.

### Dominant Microbial Community in Relation to Environmental Variables

**FIGURE 7:**
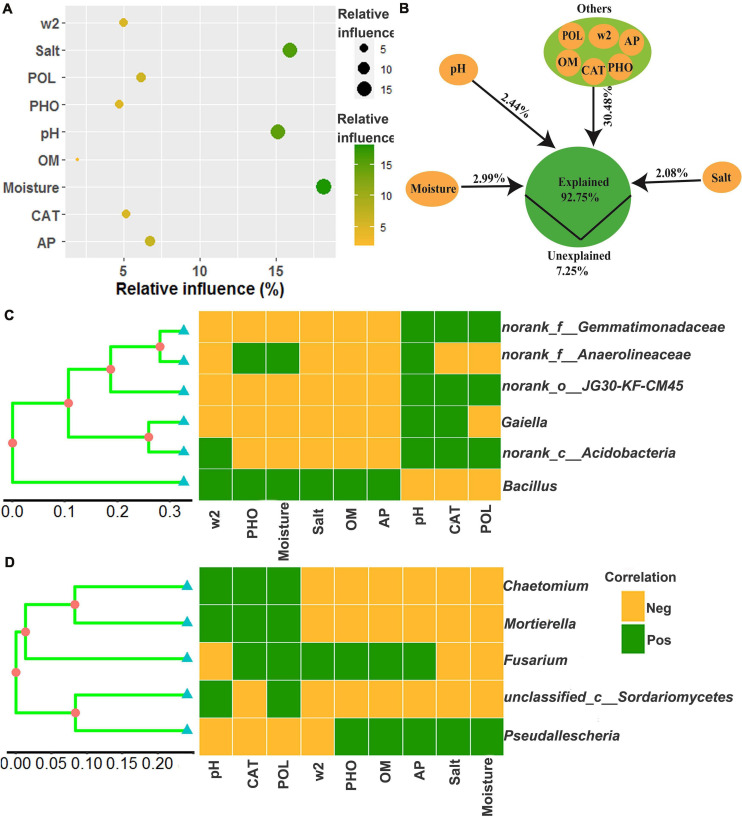
The correlation between the environmental variables and the dominant microbial community. **(A)** the relative influence of the environmental variables on the dominant microbial community; **(B)** the contribution of the environmental variables to the assembly process of the dominant microbial community; **(C,D)**, Mantel test based on the Bray-Curtis distance. W2 represents >2 mm water-stable aggregates, and CAT, POL, and PHO are catalase, polyphenol oxidase and alkaline phosphatase activities, respectively.

The relative influence of the environmental variables on the dominant microbial community using the ABT method is shown in Figure 7A. The factors examined showed a relative influence on dominant bacteria and fungi in the following order: moisture, salt, pH, AP, polyphenol oxidase activity, catalase activity, >2 mm water-stable aggregates, alkaline phosphatase activity, and OM, and the relative influences of these environmental variables were 18.16, 15.93, 15.12, 6.77, 6.14, 5.20, 5.00, 4.72, and 1.98%, respectively. The contribution of moisture, salt, pH, and other environmental variables to the dominant microbial community was illustrated with a modified VPA (Figure 7B). The complete set of all variables together explained 92.75% of the variation in the dominant microbial community of the chili rhizosphere soil, of which moisture, pH and salt contributed a larger proportion of variation than did the other soil environmental variables.

The relationships between the dominant microbial community and environmental variables were examined using a Mantel test, and the results are shown in Figures 7C,D and Supplementary Figures 1–9. There were significant associations (*R* values were between 0.3174 and 0.7165) between the dominant microbial community and the environmental variables (*p* values were less than 0.05), including norank_f__Gemmatimonadaceae and norank_o__JG30-KF-CM45 were positively correlated with pH, polyphenol oxidase activity and catalase activity, but negatively correlated with >2 mm water-stable aggregates, alkaline phosphatase activity, moisture, salt, OM, and AP; norank_f__Anaerolineaceae were positively correlated with pH, alkaline phosphatase activity and moisture, but negatively correlated with >2 mm water-stable aggregates, salt, OM, AP, polyphenol oxidase activity, and catalase activity; *Gaiella* was positively correlated with pH and catalase activity, but negatively correlated with >2 mm water-stable aggregates, alkaline phosphatase activity, moisture, salt, OM, AP, and polyphenol oxidase activity; norank_c__Acidobacteria were positively correlated with >2 mm water-stable aggregates, pH, polyphenol oxidase activity and catalase activity, but negatively correlated with alkaline phosphatase activity, moisture, salt, OM, and AP; *Bacillus* was positively correlated with >2 mm water-stable aggregates, alkaline phosphatase activity, moisture, salt, OM, and AP, but negatively correlated with pH, polyphenol oxidase activity and catalase activity; *Chaetomium* and *Mortierella* were positively correlated with pH, polyphenol oxidase activity and catalase activity, but negatively correlated with >2 mm water-stable aggregates, alkaline phosphatase activity, OM, AP, salt, and moisture; *Fusarium* was positively correlated with catalase activity, polyphenol oxidase activity, >2 mm water-stable aggregates, alkaline phosphatase activity, OM and AP, but negatively correlated with pH, salt and moisture; unclassified_c__Sordariomycetes was positively correlated with pH and polyphenol oxidase activity, but negatively correlated with catalase activity, >2 mm water-stable aggregates, alkaline phosphatase activity, OM, AP, salt, and moisture; *Pseudallescheria* was positively correlated with alkaline phosphatase activity, OM, AP, salt, and moisture, but negatively correlated with pH, catalase activity, polyphenol oxidase activity and >2 mm water-stable aggregates.

## Discussion

Knowledge of the effects of continuous cropping on different plants has accumulated rapidly over the past few years (Li et al., 2016, 2017; Fu et al., 2017; Qin et al., 2017). However, few studies have investigated the assembly process of the dominant microbial communities in the rhizosphere soil of long-term chili monocultures, particularly with regard to the determinants of abundant microbial community dynamics. Based on the analyses of environmental variables and high-throughput sequencing, the present study reveals distinct assembly processes governing the abundant subcommunities, and we also provide statistical evidence of potential environmental variables correlated with the assembly of dominant microbial communities in the rhizosphere of long-term chili monocultures.

### Long-Term Chili Monoculture Alters Rhizosphere Soil Environmental Variables

Increasing number of years of chili monoculture generated significant changes in soil nutrient composition, with an overall increase in the nutrient content. Specifically, the levels of AP, AN, and HN increased linearly with increasing duration of the chili monoculture, whereas OM, AK, and NN showed nonlinear responses. The nonlinear responses were in some cases characterized by clear thresholds, where the parameters, including OM, AK, and NN, changed dramatically in magnitude or direction of response. The increasing contents of soil available nutrients in the chili monoculture over time resulted from the high chemical fertilizer inputs (Xiong et al., 2015b; Jiao et al., 2019).

The results of the present study revealed a significant decline in soil pH with the extension of the chili monoculture time, and the long-term application of chemical fertilizers, which might be the main factors resulting in declines in soil pH (Karlen et al., 1991; Li et al., 2016; Zhao et al., 2018). Another possible cause of soil acidification in chili cropping systems is the accumulation of phenolic acids from roots (Ehrenfeld et al., 2005; Li et al., 2016; Zhao et al., 2018). Moreover, the levels of soil salt and moisture showed nonlinear responses in this study. The utilization of low-quality irrigation water can lead to the accumulation of salts in the soil, since the leaching fraction is reduced and the salts contained in the irrigation water are not sufficiently leached (Machado and Serralheiro, 2017). Under salinity stress, plant growth is inhibited due to the low water potential and ion toxicity and imbalance caused by salinity (Kaouther et al., 2012).

Soil is primarily composed of microaggregates (<0.25 mm), which bind soil organic carbon and protect it from removal by erosion, and macroaggregates (0.25–2 mm), which limit oxygen diffusion and regulate water flow (Wilpiszeski et al., 2019). Soil aggregates are heterogeneous assemblages of OM and mineral particulates (Wang et al., 2019), and the distribution and relative abundance of micro- and macroaggregates also influence a soil’s bulk properties, including the organic carbon content, water content, and niche availability (Wilpiszeski et al., 2019). In this study, we found that most soil aggregates were nonlinearly changed. For mechanically stable aggregates, macroaggregates were increased, while microaggregates showed opposite trends; for water-stable aggregates, macroaggregates and microaggregates were decreased except for the 1–2 mm water-stable aggregates, and the macroaggregates with water-stable aggregates were the best structures in the soil, and the lower the content, the worse the agglomeration and stability of soil aggregates (Zhou et al., 2020). In a previous study, OM was found to be related to the formation of soil macroaggregates (>0.25 mm) (Liu et al., 2014a), and the balance between the formation and breakdown of macroaggregates determined macroaggregate turnover, having an indirect effect on microaggregate formation (Six et al., 2004). Additionally, small microaggregates assemble into progressively larger macroaggregates held together by organomineral complexes of fungi, roots, or derived organic matter (Wilpiszeski et al., 2019).

Soil enzymes mainly originate from microorganisms and plants and are closely associated with C, N, and P cycling in soil (Fu et al., 2017; Sanchez-Hernandez et al., 2018). Soil enzyme activities have been suggested as proper indicators of soil quality and functional microbial diversity because they control key metabolic pathways in soil. They could even provide insights into the uptake of nutrients in soil as affected by land management, such as cropping systems (Li et al., 2012; Xiong et al., 2015b). In this study, soil catalase and β-glucosidase activities significantly decreased, the results were consistent with the precious study that continuous monocropping of peanut and black pepper led to a decrease in soil enzyme activities (Li et al., 2012; Xiong et al., 2015b); polyphenol oxidase activity increased at 10 years and then decreased, implying that the decomposition of benzene compound might have been hindered. The reduction of polyphenol oxidase activity could lead to the accumulation of phenolic acids in soil, potentially causing peanut autotoxicity (Liu et al., 2012). The results of present study revealed a significant decline in soil pH and enzymatic activities under long-term continuous cropping system, which might limit the chili growth in fields.

### The Determinants of the Dominant Microbial Community Dynamics

Environmental change mediates the dynamic balance of microbial communities and then influences the associated ecosystem function (Smith et al., 2016). Most studies have focused on the effects of continuous cropping on community composition, regardless of the determinants of the microbial community dynamics (Li et al., 2016, 2017; Fu et al., 2017; Qin et al., 2017). Here, we provide a more in-depth understanding of whether environmental variables correlate with the assembly process of dominant microbial communities in the rhizosphere of long-term chili monocultures.

In this study, at the phylum level, the Acidobacteria and Firmicutes were the two dominant phyla (Figures 4A,D), and Acidobacteria were the most common phyla in different agricultural systems (Xiong et al., 2015a,b). Firmicutes corresponded with soil-borne disease suppression (Xiong et al., 2015b), increasing continuous cropping duration was positively correlated with the relative abundance of Firmicutes (Figure 4D, *R* = 0.91, *p* < 0.0001), and the result was contrary to previous studies that Firmicutes were less abundant in disease conducive soils. The reason for the above result in this study may be that the plant recruitment of beneficial microbes to suppress soil-borne pathogen (Shi et al., 2019; Liu et al., 2021).

Many soil-borne diseases were caused by *Fusarium*, which contains many pathogenic species; thus, an increase in *Fusarium* abundance is likely to lead to plant disease (Xiong et al., 2015b; Gu et al., 2020). In this study, the relative abundance of *Fusarium* (Figure 6C) decreased with continuous cropping for 5 years, then increased, and 10 years and 20 years showed no significant change with 1 year in the chili rhizosphere soil samples, and the reason for this result may be the change of relative abundance of beneficial microbes. *Bacillus* is one of the most famous biocontrol bacteria and it is widely used in the biological control of various plant diseases (Shi et al., 2019) and increasing continuous cropping duration was positively correlated with the relative abundance of *Bacillus* (Figure 5B, *R* = 0.96, *p* < 0.0001). Moreover, based on ABT, VPA, and Mantel tests, *Bacillus* was positively correlated with >2 mm water-stable aggregates, alkaline phosphatase activity, moisture, salt, OM, and AP, but negatively correlated with pH, polyphenol oxidase activity and catalase activity. In a previous study, *Bacillus amyloliquefaciens* FZB42 improved the P status (Mpanga et al., 2020), and the inoculation of *Bacillus* isolates significantly increased the activity of alkaline phosphatase (Ramesh et al., 2011). Correlation studies of other dominant microbes and the environmental variables in this study have not been reported. Therefore, focused on the changes of the soil dominant microbial community in the field of chili growth promotion and disease resistance were important for chili production.

In previous studies, salt was identified as one of the most powerful environmental factors structuring microbial communities in aquatic and soil environments (Rath and Rousk, 2015). Soil moisture and other soil variables were closely related to the microbial community composition and biomass at the regional scale and in laboratory incubation (Ma et al., 2015; Zhou et al., 2017). Additionally, pH is widely recognized as an important factor shaping soil microbial composition. Based on ABT analysis and VPA as well as the Mantel test, the determinants of the dominant microbial community dynamics were moisture, salt and pH in this study. Globally, pH tends to decrease with N addition (Zhang X. et al., 2014; Tian and Niu, 2015), and in this study, the observed decresed in pH, increase in N, and even the increased N-cycling enzyme activity and decreased C-cycling enzyme seem to tell a story of abiotic nitrogen accumulation in these systems, which may be a driving force shaping the microbial community. Moisure and N are the two key factors that limit plant productivity in arid and semiarid grassland ecosystems, and the changes in the levels of these two factors profoundly influence the biodiversity and ecosystem functioning. N addition and watering may have combined effects on soil microbial communities (Zhang X. et al., 2014). N addition and water addition tends to reduce the relative abundance of Acidobacteria, and the possible mechanism underlying the responses of bacterial phyla to water addition in grasslands was that watering could increase nitrogen availability by stimulating the mineralization of soil organic matter (She et al., 2018), and the possible mechanism underlying the responses of bacterial phyla to N addition was that the decrease of soil pH was unlikely the mechanism of N addition effects on the decline of bacterial biomass because Acidobacteria was adapted to low pH conditions (Wang et al., 2018), however, Acidobacteria was negtivly correlation with pH in this study, it could be a difference in soil type (Zhang Y. et al., 2014); Firmicutes are suitable for the bioremediation in hypersaline conditions, and the remarkably higher Firmicutes abundance find in salinized farmland could be conducive to the improvement of soil salinization (Cheng et al., 2018), and also N addition led to an increase in the relative abundance of Firmicutes (Zhang X. et al., 2014), and the increase of Firmicutes may be the result of a combination of these two factors. Thus, exploring sustainable agricultural measures to improve soil pH and N, soil enzymatic activities and soil microbes are extremely important for the chili production and will be the focus of our future research.

## Conclusion

The time-scale experiments of chili continuous cropping were used to assess the changes of soil environmental variables, and assembly of dominant microbial communities, as well as provides a new view on the improvement of soil continuous cropping barrier. In this study, long-term chili monoculture altered the rhizosphere soil environmental variables and changed the assembly process of the dominant microbial community. In addition, plant recruitment of beneficial microbes (*Firmicutes* and *Bacillus*) to suppress soil-borne pathogen (*Fusarium*), and the drivers of the dominant microbial community assembly in rhizosphere soil were soil moisture, abiotic nitrogen, pH, and salt.

## Data Availability Statement

The data of 16S rRNA and ITS gene sequence were deposited to the National Center for Biotechnology Information (NCBI) under the project accession number PRJNA715611.

## Author Contributions

ZW made substantial contributions to the design, the acquisition, analysis, and interpretation of data for the work. WC and XG performed the experiment and drafted the work. QG and XT revised it critically for important intellectual content. All authors contributed to the article and approved the submitted version.

## Conflict of Interest

The authors declare that the research was conducted in the absence of any commercial or financial relationships that could be construed as a potential conflict of interest.

## Footnotes

1 http://drive5.com/uparse/

2 http://rdp.cme.msu.edu/
