# Supplementary material for: Long-Term Chili Monoculture Alters Environmental Variables Affecting the Dominant Microbial Community in Rhizosphere Soil
Source: Front Microbiol. 2021 Jul 1;12:681953. doi: 10.3389/fmicb.2021.681953 (PMC8281244; doi:10.3389/fmicb.2021.681953)
Supplement: Supplementary file 1 [file Data_Sheet_1.docx]

**Supplementary Table 1.** Effect of long-term chili monoculture on the chemical properties of rhizosphere soil

| Time (year) | pH | AN (mg/kg) | AP (mg/kg) | HN (mg/kg) | Salt (%) | Moisture (%) | AK (mg/kg) | OM (mg/kg) | NN (mg/kg) |
| --- | --- | --- | --- | --- | --- | --- | --- | --- | --- |
| 1 | 8.27±0.16 a | 5.26±0.54 b | 30.43±8.59 c | 81.30±7.4 c | 0.10±0.01 c | 14.5±0.10 d | 266.25±16.25 c | 16.0±0.01 d | 55.82±3.56 c |
| 5 | 7.78±0.19 b | 5.93±0.23 ab | 144.03±40.28 b | 171.85±27.75 b | 0.12±0 b | 22.6±0.4 b | 334±24 b | 2.76±0.15 c | 71.55±1.46 b |
| 10 | 7.66±0.07 b | 6.70±0.41 a | 313.67±25.78 a | 224.23±17.03 a | 0.12±0.01 b | 19.73±0.35 bc | 444±10 a | 4.48±0.04 b | 81.57±9.56 b |
| 20 | 7.56±0.10 b | 6.71±0.15 a | 341.57±36.61 a | 248.83±11.3 a | 0.16±0 a | 28.6±0.85 a | 441.5±12.5 a | 5.35±0.35 a | 157.66±6.1 a |

Note: The different letters indicate significant differences; organic matter (OM), available phosphorus (AP), available potassium (AK), hydrolyzable nitrogen (HN), ammonium nitrogen (AN) and nitrate nitrogen (NN).

**Supplementary Table 2.** Effect of long-term chili monoculture on the aggregates of rhizosphere soil

| Time (year) | Water-stable aggregate (%) | | | | Mechanically stable aggregate (%) | | | |
| --- | --- | --- | --- | --- | --- | --- | --- | --- |
|  | >2 mm | 1-2 mm | 0.25-1 mm | < 0.25 mm | >2 mm | 1-2 mm | 0.25-1 mm | < 0.25 mm |
| 1 | 13.69±4.91 b | 10.71±0.99 b | 20.24±0.06 b | 46.49±0.26 a | 40.37±0.26 a | 9.02±0.18 c | 32.92±1.36 a | 16.0±0.70 a |
| 5 | 11.90±0.78 b | 10.88±2.83 b | 20.65±0.5 b | 44.99±10.21 ab | 37.79±0.12 b | 9.34±0.03 b | 28.76±1.02 b | 12.34±1.07 b |
| 10 | 31.13±4.95 a | 17.86±0.96 a | 23.10±1.08 a | 28.85±0.28 c | 36.53±1.15 b | 10.40±0.02 a | 20.34±2.31 c | 8.46±0.07 c |
| 20 | 26.64±2.71 a | 14.13±0.99 ab | 22.48±0.75 a | 32.25±1.98 bc | 33.94±0.64 c | 10.22±0.06 a | 23.55±0.44 c | 6.89±0.32 c |

Note: The different letters indicate significant differences

**Supplementary Table 3.** Effect of long-term chili monoculture on the enzyme activities of rhizosphere soil

| Time (year) | Catalase (U/g) | β-glucosidase (U/g) | Nitrate reductase (U/g) | Polyphenol oxidase (U/g) | Urease (U/g) | Alkaline phosphatase (U/g) |
| --- | --- | --- | --- | --- | --- | --- |
| 1 | 1692.46±17.40 a | 11.79±0.30 a | 15.42±0.63 c | 14.61±0.09 b | 451.89±29.70 a | 13.54±0.18 d |
| 5 | 485.50±2.35 d | 6.84±0.88 b | 21.08±0.90 b | 14.21±0.09 c | 423.64±12.47 a | 13.98±0.24 c |
| 10 | 1445.64±34.11 b | 6.16±1.08 b | 19.53±0.99 bc | 18.53±0.02 a | 404.16±2.22 a | 17.58±0.03 a |
| 20 | 1006.31±8.27 c | 5.44±0.65 b | 46.57±3.14 a | 13.61±0.14 d | 412.86±37.26 a | 15.25±0.02 b |

Note: The different letters indicate significant differences

**Supplementary Table 4.** Effect of long-term chili monoculture on the relative abundance of dominant microbes at the phyla level

| Time (year) | *Acidobacteria* (%) | *Actinobacteria* (%) | *Chloroflexi* (%) | *Firmicutes* (%) | *Gemmatimonadetes* (%) | *Ascomycota* (%) | *Zygomycota* (%) |
| --- | --- | --- | --- | --- | --- | --- | --- |
| 1 | 15.46±3.10 a | 23.83±2.26 a | 18.52±3.33 a | 4.56±1.58 b | 3.96±0.38 a | 77.02±5.64 a | 14.57±5.06 a |
| 5 | 7.01±1.38 b | 27.03±3.91 a | 14.15±1.50 a | 9.63±2.29 ab | 3.28±0.39 a | 89.89±4.67 a | 6.30±3.76 a |
| 10 | 8.67±4.35 ab | 20.33±3.32 a | 14.19±2.84 a | 14.67±0.39 a | 3.23±0.37 a | 89.96±9.41 a | 3.58±0.78 a |
| 20 | 9.24±2.24 ab | 20.23±1.62 a | 13.52±1.69 a | 18.74±3.32 a | 3.06±0.35 a | 95.30±3.09 a | 4.03±2.95 a |

Note: The different letters indicate significant differences

**Supplementary Table 5.** Effect of long-term chili monoculture on the relative abundance of dominant bacteria at the genus level

| Time (year) | *Bacillus* (%) | *Gaiella* (%) | *norank_c__ Acidobacteria* (%) | *norank_f__ Anaerolineaceae* (%) | *norank_f__ Gemmatimonadaceae* (%) | *norank_o__ JG30-KF-CM45* (%) |
| --- | --- | --- | --- | --- | --- | --- |
| 1 | 1.96±0.47 c | 1.75±0.14 a | 10.62±2.33 a | 3.20±0.56 a | 2.65±0.28 a | 4.13±0.37 a |
| 5 | 3.58±0.76 c | 1.44±0.01 b | 5.06±1.01 b | 3.41±1.10 a | 1.55±0.07 b | 3.51±0.43 ab |
| 10 | 8.75±0.89 b | 1.27±0.03 b | 8.64±1.05 a | 3.25±1.07 a | 1.71±0.13 b | 3.05±0.34 b |
| 20 | 12.25±1.16 a | 1.41±0.03 b | 7.06±1.80 a | 3.25±0.88 a | 1.51±0.26 b | 2.85±0.18 b |

Note: The different letters indicate significant differences

**Supplementary Table 6.** Effect of long-term chili monoculture on the relative abundance of dominant fungi at the genus level

| Time (year) | *Chaetomium* (%) | *Fusarium* (%) | *Mortierella* (%) | *Pseudallescheria* (%) | *unclassified_c_Sordariomycetes* (%) |
| --- | --- | --- | --- | --- | --- |
| 1 | 6.41±2.14 a | 2.16±0.01 ab | 17.19±0.32 a | 1.13±0.09 c | 2.07±0.55 c |
| 5 | 4.57±1.67 a | 1.43±0.07 b | 6.31±0.86 b | 7.90±1.26 a | 9.21±0.27a |
| 10 | 3.22±0.22 a | 2.71±0.17 a | 2.29±0.09 c | 2.77±1.17 bc | 5.59±1.14 b |
| 20 | 1.36±0.46 b | 2.08±0.91 a | 2.07±1.11 c | 6.48±2.37 ab | 1.32±0.12 c |

Note: The different letters indicate significant differences


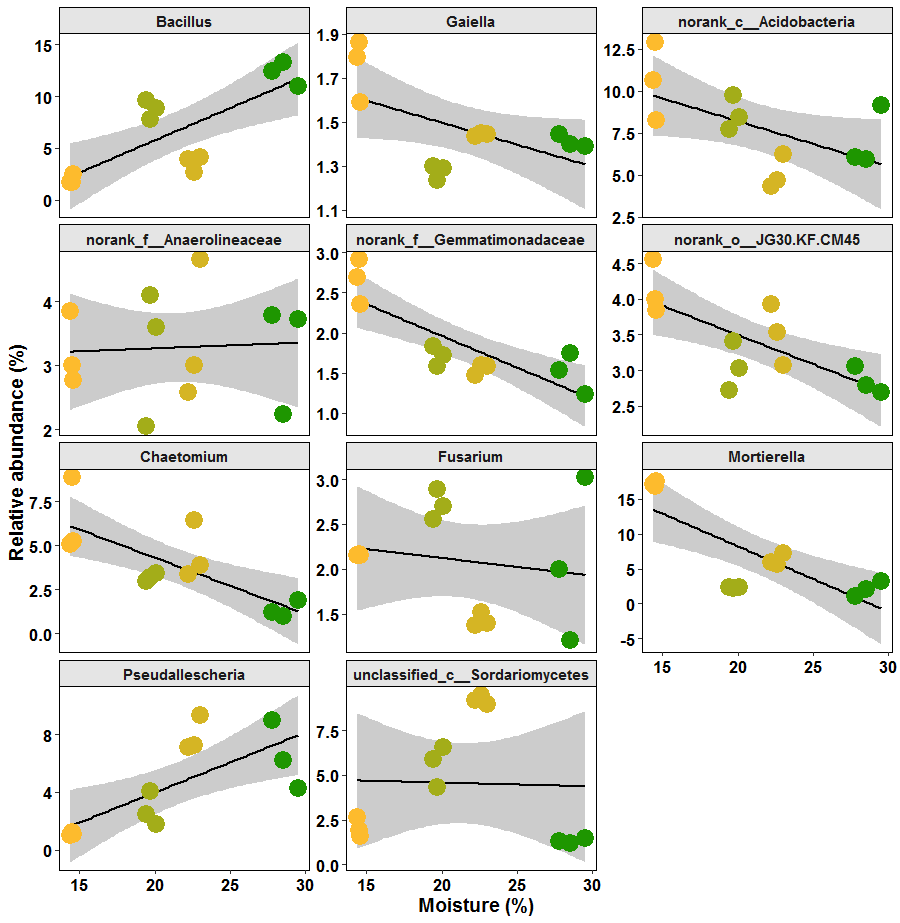


**Supplementary Figure 1.** The relationships between the dominant microbial community and moisture were examined using a Mantel test.

**
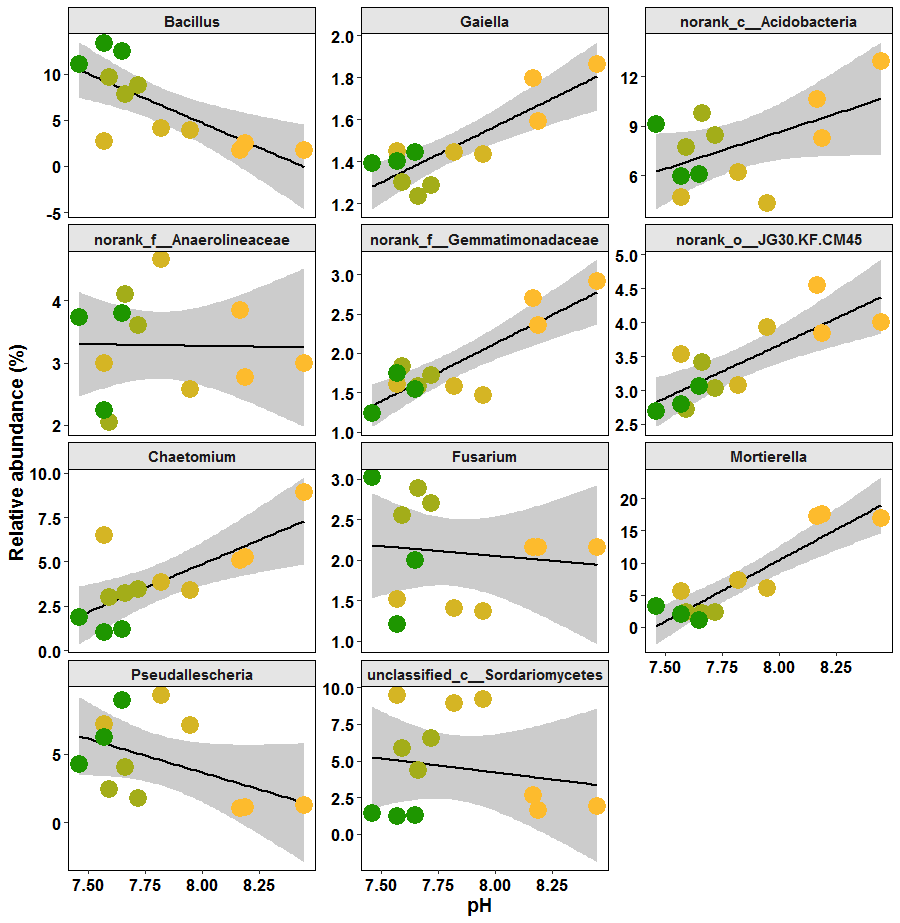
**

**Supplementary Figure 2.** The relationships between the dominant microbial community and pH were examined using a Mantel test.


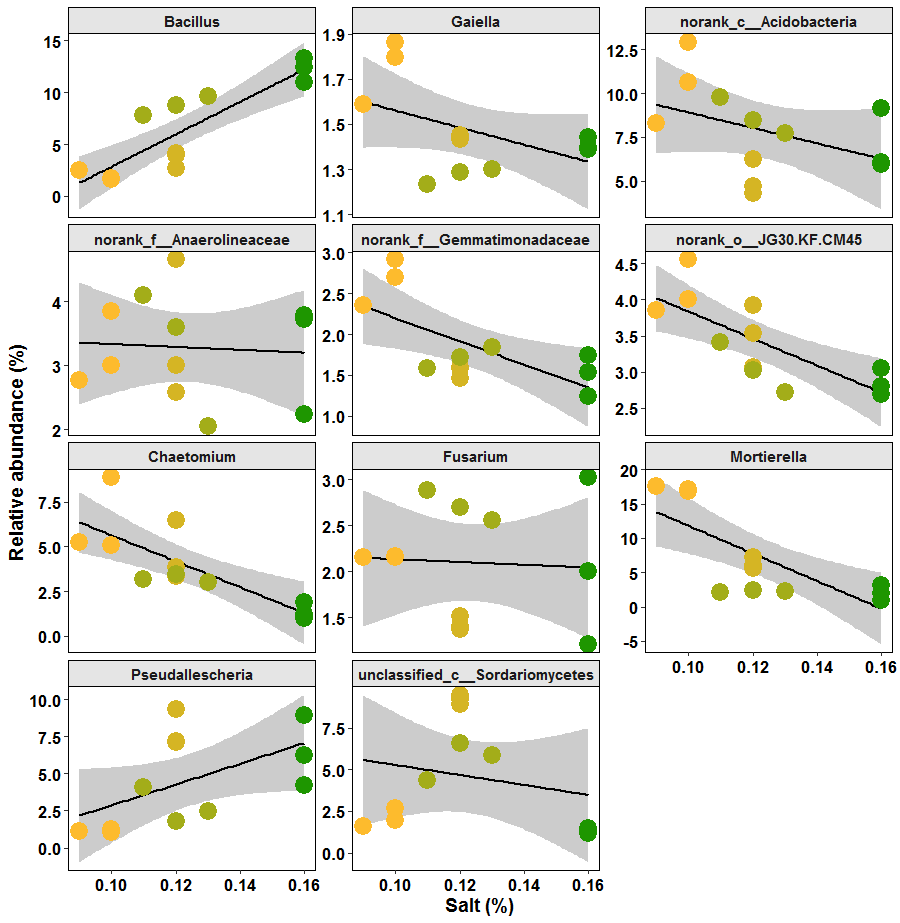


**Supplementary Figure 3.** The relationships between the dominant microbial community and salt were examined using a Mantel test.


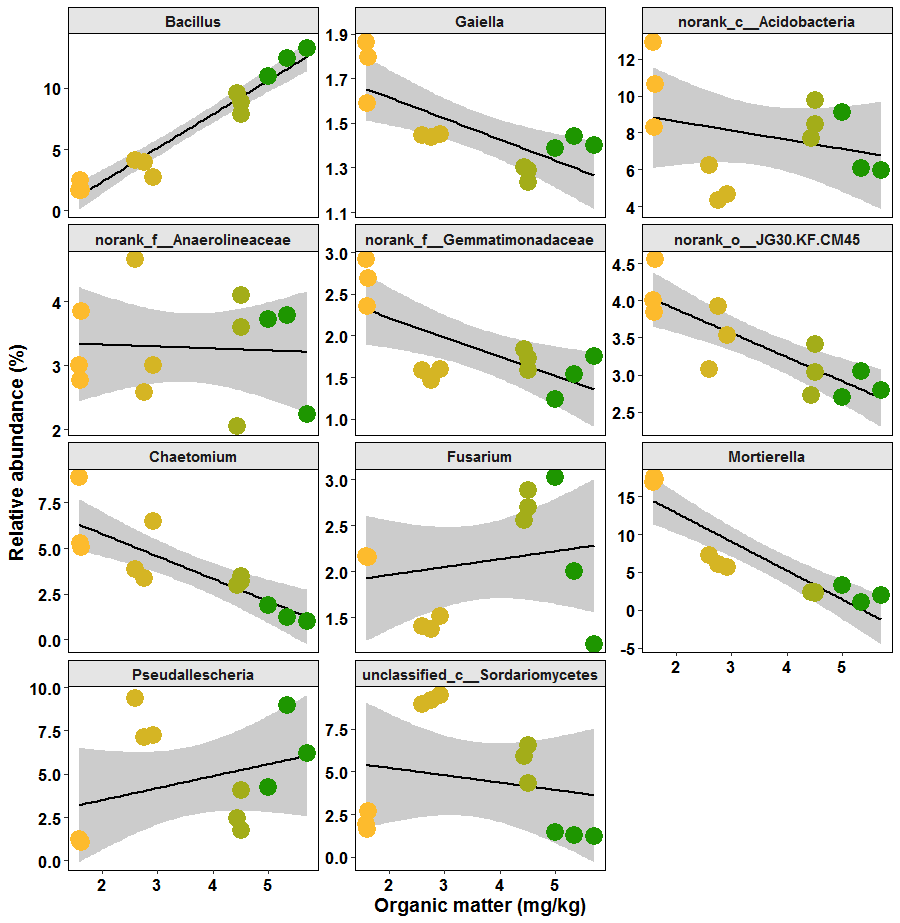


**Supplementary Figure 4.** The relationships between the dominant microbial community and organic matter were examined using a Mantel test.


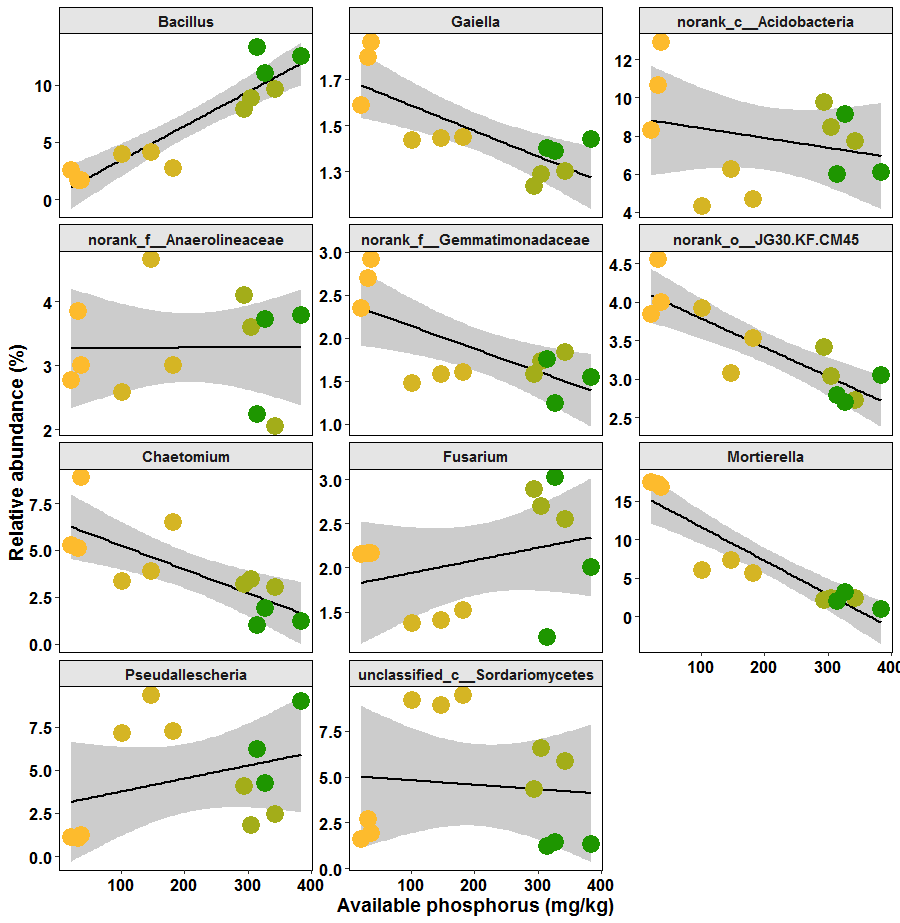


**Supplementary Figure 5.** The relationships between the dominant microbial community and available phosphorus were examined using a Mantel test.


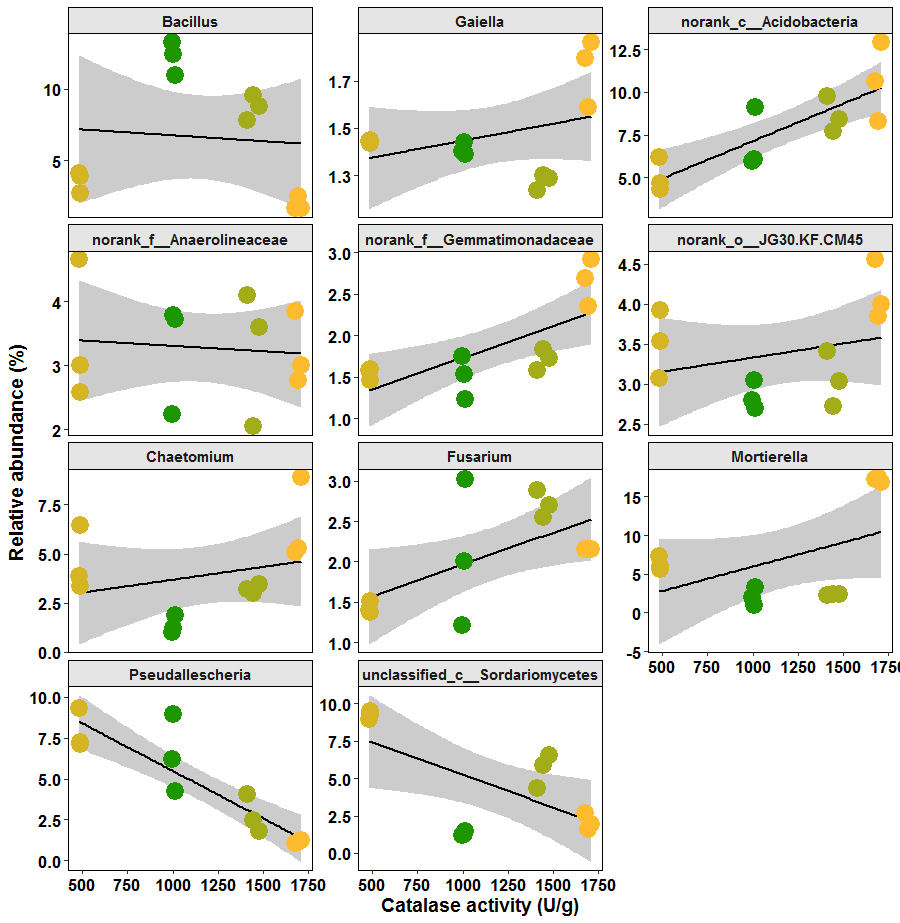


**Supplementary Figure 6.** The relationships between the dominant microbial community and catalase activity were examined using a Mantel test.


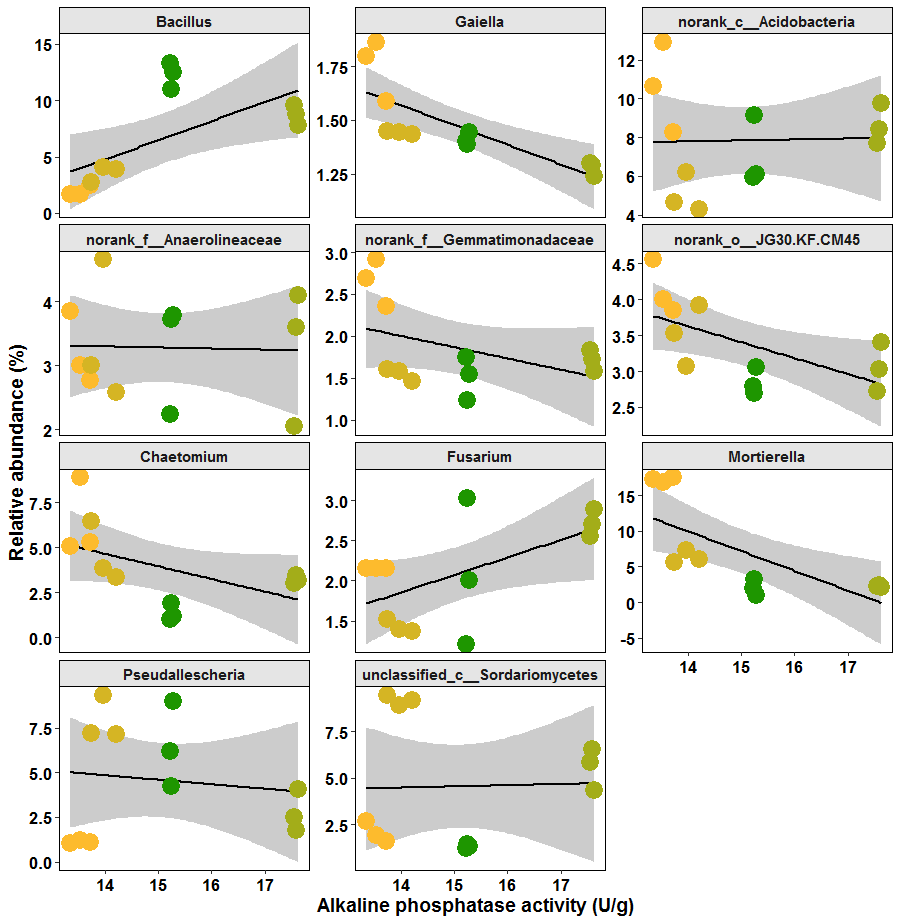


**Supplementary Figure 7.** The relationships between the dominant microbial community and alkaline phosphatase activity were examined using a Mantel test.


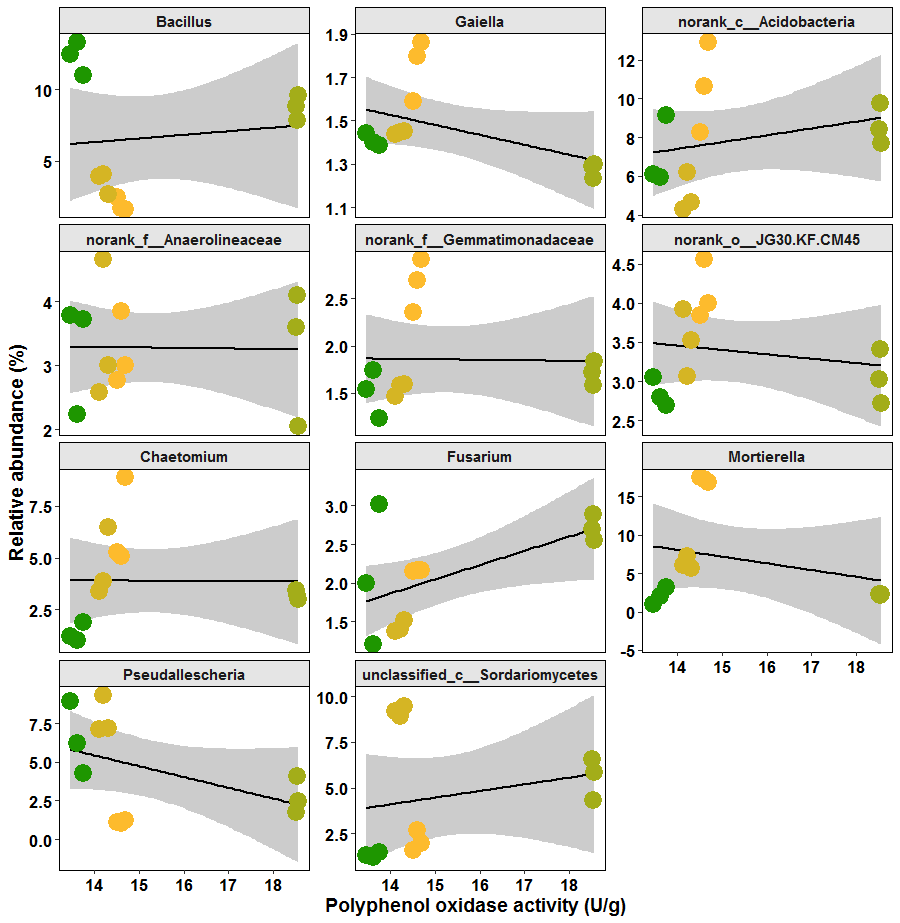


**Supplementary Figure 8.** The relationships between the dominant microbial community and polyphenol oxidase activity were examined using a Mantel test.


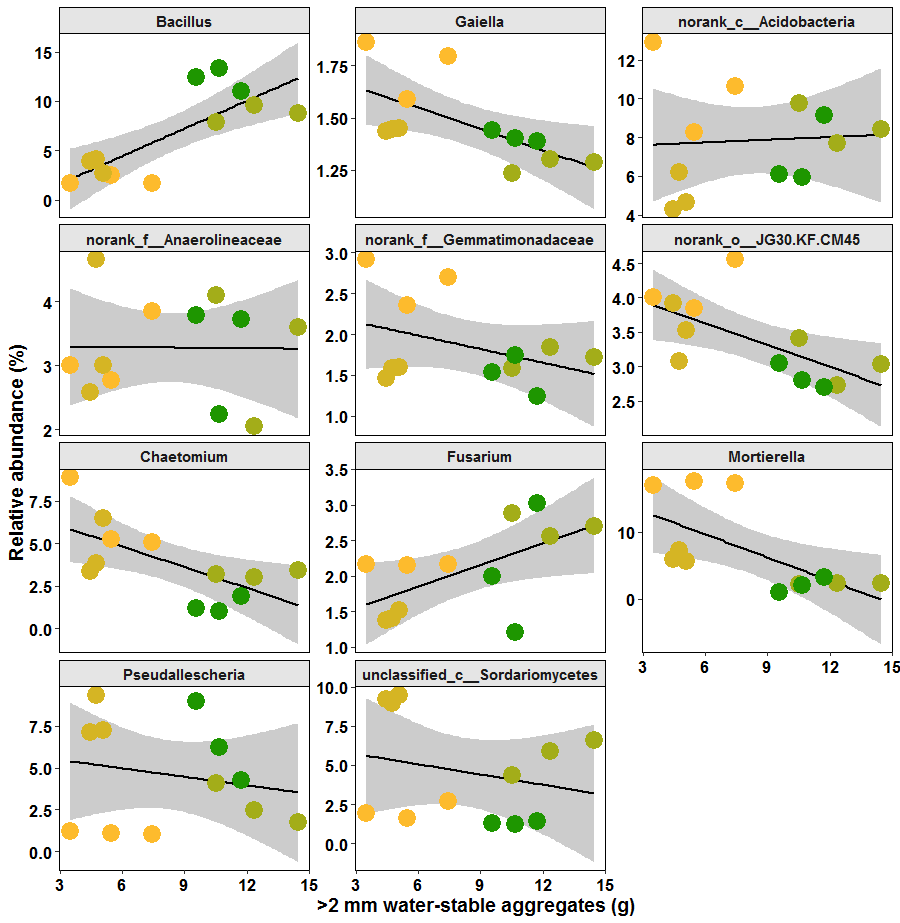


**Supplementary Figure 9.** The relationships between the dominant microbial community and > 2 mm water-stable aggregates were examined using a Mantel test.
